# Gain-Enhancement Strategy for C-Band Polymer Waveguide Amplifiers via Dual-Wavelength Pumping

**DOI:** 10.3390/polym18212732

**Published:** 2026-11-03

**Authors:** Changlong Li, Hong Liao, Guanlin Li, Zheng Wang, Kezhi Zheng, Fei Wang, Daming Zhang

**Affiliations:** 1 State Key Laboratory of Integrated Optoelectronics, College of Electronic Science and Engineering, College of Integrated Circuits, Jilin University, Changchun 130012, Chinaleeplumcan@163.com (G.L.); zhangdm@jlu.edu.cn (D.Z.); 2 Key Laboratory of Atomic and Subatomic Structure and Quantum Control (Ministry of Education), School of Physics, Guangdong Provincial Key Laboratory of Quantum Engineering and Quantum Material, Guangdong-Hong Kong Joint Laboratory of Quantum Matter, Frontier Research Institute for Physics, South China Normal University, Guangzhou 510006, China; 2023022116@m.scnu.edu.cn

**Keywords:** lanthanide-doped nanoparticles, energy transfer, polymer waveguide amplifier, dual-wavelength pumping, gain enhancement

## Abstract

Optical waveguide amplifiers are vital for on-chip loss compensation in photonic integrated circuits (PICs). Although C-band waveguide amplifiers have been widely studied, achieving high gain remains a considerable challenge. In this work, we present a gain-enhancement strategy based on core-shell nanoparticles (NPs) co-doped with multiple rare-earth ions (NaYbF_4_:Er^3+^, Ce^3+^@NaYF_4_:Yb^3+^, Nd^3+^). Efficient energy transfer from Nd^3+^ to Yb^3+^ ions significantly boosts the downconversion emission of Er^3+^ ions. When incorporated into a polymer waveguide amplifier, these NPs enable a maximum measured relative gain of 11.62 dB at 1535 nm in a 0.8 cm-long device under dual-wavelength (808 and 976 nm) pumping, which is 3.18 dB higher than the highest relative gain obtained under 976 nm single-wavelength pumping. This approach provides a route to enhanced C-band amplification and offers a strategy for designing compact, multi-wavelength-pumped photonic devices.

## 1. Introduction

The development of erbium-doped waveguide amplifiers (EDWAs) was motivated by the outstanding performance of erbium-doped fiber amplifiers (EDFAs), which have been widely deployed since the 1980s in long-distance optical fiber communication systems. EDFAs are particularly valued for their high gain, broad bandwidth, and low loss, especially in the C-band (1530–1565 nm) communication window [1]. Building on these benefits, EDWAs have emerged as critical components in photonic integrated circuits (PICs), enabling seamless integration with other photonic devices. In particular, EDWAs offer a reliable solution for compensating optical losses in the C-band, thereby facilitating effective amplification within integrated photonic systems.

Erbium-doped waveguide amplifiers (EDWAs) have been successfully fabricated using a range of host materials, including Si_3_N_4_ [2], LiNbO_3_ [3,4,5,6,7,8,9,10,11], Al_2_O_3_ [12,13,14,15,16,17], Ta_2_O_5_ [18], TeO_2_ [19], and polymer [20,21], all of which have demonstrated promising optical amplification performance. Recent studies have reported several key advancements in this field. For instance, a 50 cm long Er^3+^:Si_3_N_4_ waveguide amplifier achieved an optical gain of 30 dB at 1550 nm when excited by a 1480 nm semiconductor laser [2]. In a similar vein, a 1.2 cm long Er^3+^:Ta_2_O_5_ waveguide amplifier, structured in a double-layer configuration, reached a maximum internal net gain of approximately 10.58 dB/cm at 1533 nm under excitation by a 980 nm semiconductor laser [18]. Furthermore, Bao et al. demonstrated a high-power EDWA based on thin-film lithium niobate, with a power conversion efficiency of 52% and a maximum on-chip output power of 113 mW, achieved using the photolithography-assisted chemo-mechanical etching (PLACE) technique [11]. Among the various host materials, polymer-based hosts have garnered significant attention due to their outstanding properties, including low production costs, ease of processing, and excellent compatibility with a wide range of substrates. These attributes make polymers highly promising for photonic integration, particularly as a compelling platform for PICs, especially for optical loss compensation. Despite these promising developments, several challenges remain. Key factors limiting the commercialization of EDWAs include the need for further improvements in gain performance, output power, and transmission efficiency, as well as the successful integration with photonic chips. Nd^3+^-sensitized nanoparticles (NPs) have been investigated extensively for luminescence applications, establishing excitation near 800 nm and energy transfer involving Nd^3+^ and Yb^3+^ ions [22,23,24,25]. The representative NPs-based polymer waveguide devices summarized in Table 1 employ single-wavelength pumping. Here, the additional Nd^3+^-mediated excitation pathway enables combined 808 and 976 nm pumping, and its contribution to optical signal amplification is experimentally evaluated at 1535 nm.

In this work, we extend the Nd^3+^ sensitization strategy to polymer waveguide amplifiers by introducing NaYbF_4_:Er^3+^, Ce^3+^@NaYF_4_:Yb^3+^, Nd^3+^ core-shell NPs as the gain medium. The NPs were synthesized using a co-precipitation method, with Nd^3+^ and Yb^3+^ ions serving as sensitizers, Er^3+^ ions as emitters, and Ce^3+^ ions as deactivators. Photoluminescence measurements reveal enhanced Er^3+^ emission under dual-wavelength (808 and 980 nm) excitation compared with 980 nm single-wavelength excitation, indicating the contribution of Nd^3+^ sensitization to the 1.5 µm emission. By incorporating these NPs into a polymer waveguide amplifier, the highest measured relative gain at 1535 nm reached 11.62 dB in a 0.8 cm-long device under dual-wavelength (808 and 976 nm) pumping, which was 3.18 dB higher than the highest measured relative gain under 976 nm-only pumping.

## 2. Results

**Figure 1:**
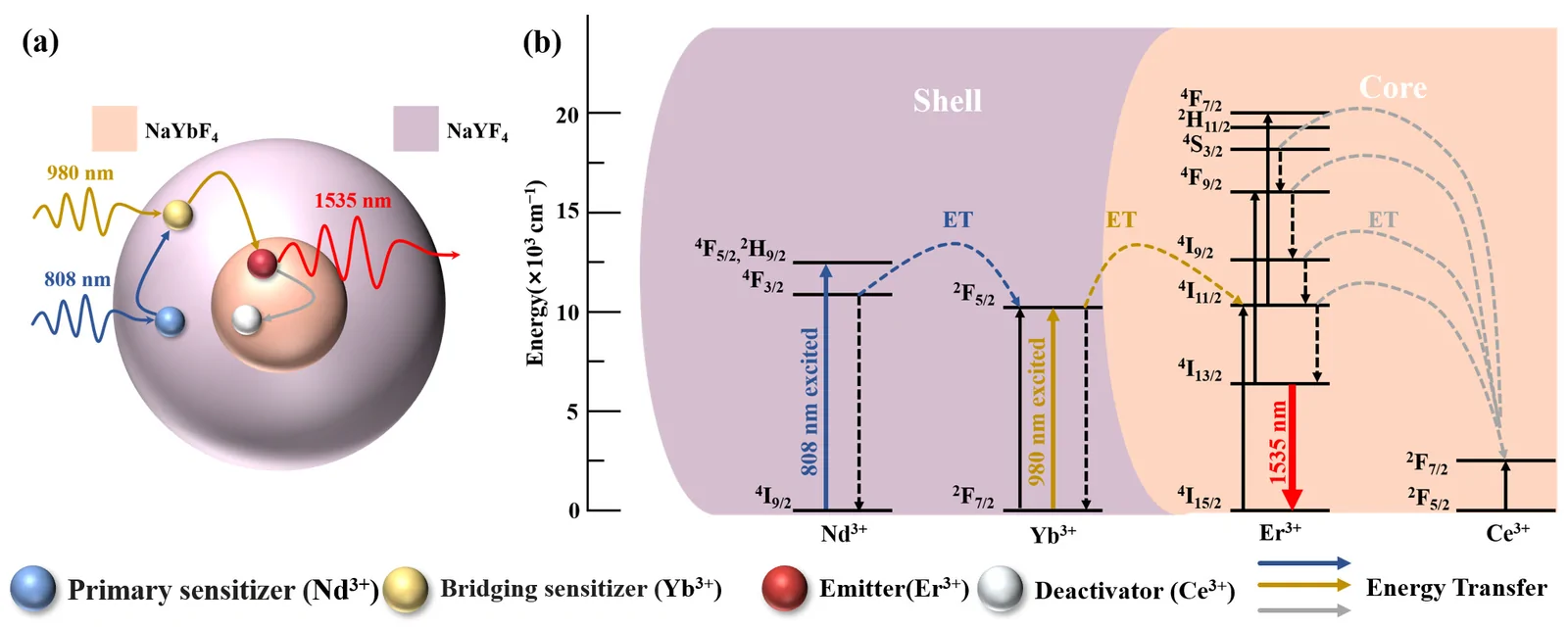
(**a**) Schematic structure of NaYbF_4_:2%Er^3+^, 2%Ce^3+^@NaYF_4_:20%Yb^3+^, 20%Nd^3+^ core–shell nanoparticles (NPs). (**b**) Energy-level diagrams of Nd^3+^, Yb^3+^, Er^3+^, and Ce^3+^ ions, illustrating the cascade sensitization mechanism under 808 nm and 980 nm laser excitation.

In our design, an Nd^3+^-Yb^3+^ dual-sensitization fluoride system was employed to enhance the downconversion (DC) emission of Er^3+^ ions. The core-shell design builds on the NaYbF_4_:2%Er^3+^, 2%Ce^3+^@NaYF_4_ architecture reported by Zhong et al. [28]. NaYbF_4_ was selected as the core host to provide a high density of Yb^3+^ sensitizers for 980 nm absorption and energy transfer to Er^3+^. NaYF_4_ was selected as the shell host to allow the Nd^3+^ and Yb^3+^ doping concentrations to be adjusted while retaining the Er^3+^/Ce^3+^-doped NaYbF_4_ core composition. As shown in Figure 1a,b, the primary sensitizer Nd^3+^ absorbs 808 nm photons and is excited from the ground state ^4^I_9/2_ to the excited state ^4^F_5/2_ level due to its strong optical absorption around 800 nm. Following non-radiative relaxation to the Nd^3+ 4^F_3/2_ level, the excitation energy is transferred to neighboring Yb^3+^ ions [23,24]. These Yb^3+^ ions, acting as bridging sensitizers, subsequently transfer the excitation energy to neighboring Er^3+^ ions, enabling emission near 1.5 μm for telecommunication applications. To facilitate this energy transfer while reducing direct Nd^3+^-Er^3+^ interactions, Nd^3+^ and Yb^3+^ were incorporated into the shell, whereas Er^3+^ was located in the Yb-rich core. This spatial separation is intended to limit potential back-energy transfer from excited Er^3+^ to Nd^3+^, while Yb^3+^ in the shell mediates energy transport across the core-shell interface toward Er^3+^ [22,25]. Therefore, we hypothesized that simultaneous pumping at 808 and 980 nm could further enhance the 1.5 μm emission by activating both excitation pathways. To further improve the DC efficiency of Er^3+^, Ce^3+^ ions were introduced as codopants into the core. The energy splitting between the ^2^F_5/2_ and ^2^F_7/2_ levels of Ce^3+^ is approximately 2300 cm^−1^, while that associated with the Er^3+ 4^I_11/2_ → ^4^I_13/2_ transition is approximately 3700 cm^−1^. As previously reported, phonon-assisted cross relaxation between Er^3+^ and Ce^3+^ facilitates the depopulation of the Er^3+ 4^I_11/2_ level and increases the population of the ^4^I_13/2_ level. This process suppresses competing upconversion and favors DC emission near 1.5 µm [28].

**Figure 2:**
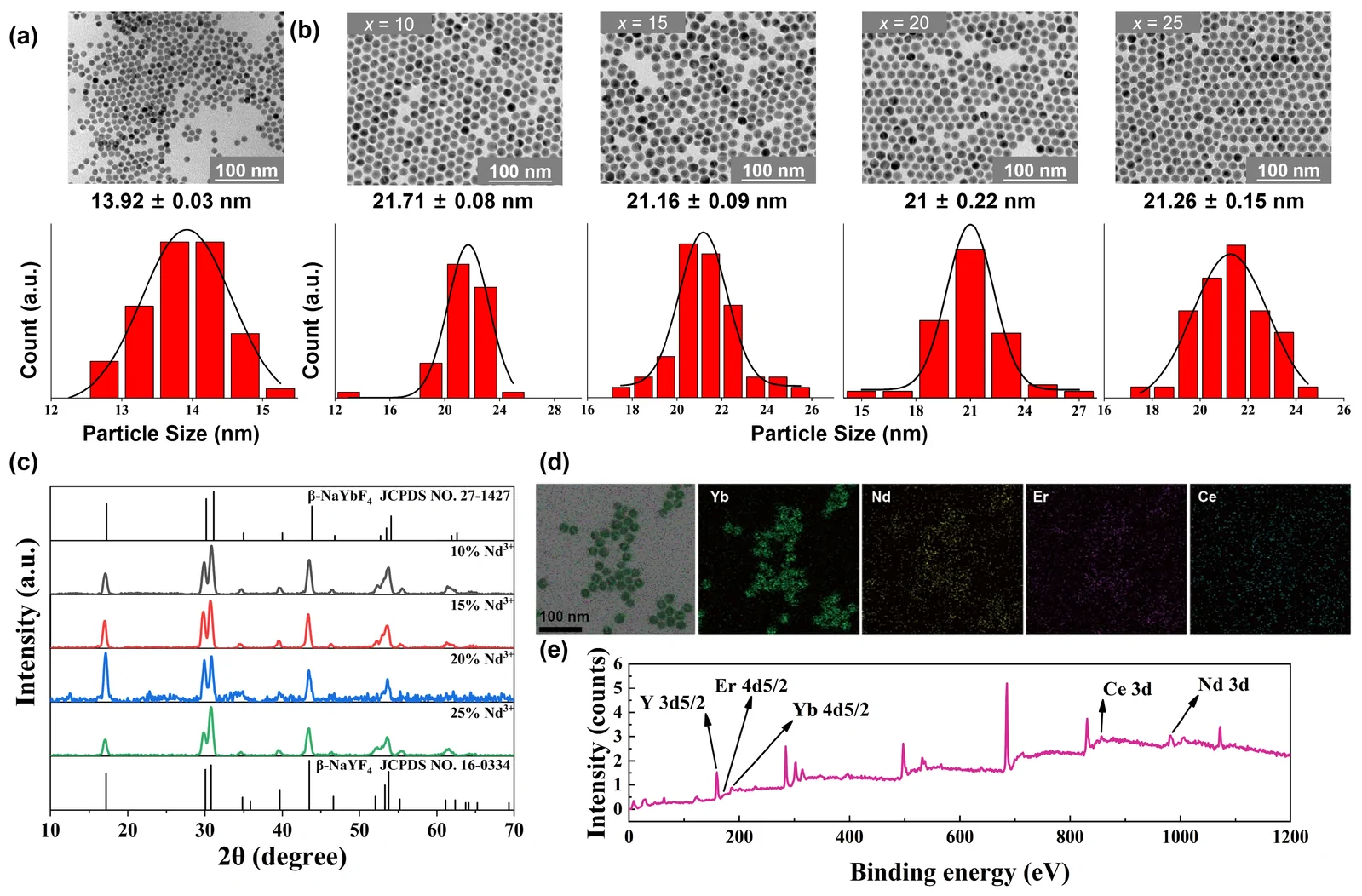
Transmission electron microscopy (TEM) images and corresponding size distributions of (**a**) NaYbF_4_:2%Er^3+^, 2%Ce^3+^ core NPs and (**b**) NaYbF_4_:2%Er^3+^, 2%Ce^3+^@NaYF_4_:20%Yb^3+^, *x*%Nd^3+^ (*x* = 10, 15, 20, and 25) core–shell NPs with varying Nd^3+^ concentrations in the shell. (**c**) X-ray diffraction (XRD) patterns of the core-shell NPs with different Nd^3+^ concentrations. The standard diffraction patterns of hexagonal β-NaYbF4 (JCPDS No. 27-1427) and β-NaYF_4_ (JCPDS No. 16-0334) are provided for comparison. (**d**) TEM-based energy-dispersive X-ray (EDX) elemental maps and (**e**) X-ray photoelectron spectroscopy (XPS) survey spectrum of NaYbF_4_:2%Er^3+^, 2%Ce^3+^@NaYF_4_:20%Yb^3+^, 20%Nd^3+^ core-shell NPs.

To investigate the effect of Nd^3+^ doping concentration, we synthesized four batches of NaYbF_4_:2%Er^3+^, 2%Ce^3+^@NaYF_4_:20%Yb^3+^, *x*%Nd^3+^ core-shell NPs (*x* = 10, 15, 20, and 25) using a co-precipitation method. The morphology and size distribution of the core-shell NPs were characterized by transmission electron microscopy (TEM). As shown in Figure 2a,b, the core–shell NPs exhibited uniform spherical morphology with average diameters of approximately 21.0–21.7 nm, compared with approximately 13.92 nm for the core NPs. This increase in diameter corresponds to an estimated shell thickness of approximately 3.5–4 nm. As shown in Figure 2c, the X-ray diffraction (XRD) patterns of the NaYbF_4_:2%Er^3+^, 2%Ce^3+^@NaYF_4_:20%Yb^3+^, *x*%Nd^3+^ (*x* = 10, 15, 20, and 25) NPs are in good agreement with the standard reference patterns of NaYbF_4_ and NaYF_4_, supporting the formation of the expected crystalline phases. TEM-based energy-dispersive X-ray (EDX) elemental maps of the selected NaYbF_4_:2%Er^3+^, 2%Ce^3+^@NaYF_4_:20%Yb^3+^, 20%Nd^3+^ core–shell NPs are presented in Figure 2d. These maps provide qualitative information on the elemental distribution. However, individual identification of low-concentration lanthanide dopants, particularly Er and Ce, remains challenging due to their similar characteristic X-ray emission energies and limited signal intensity. In addition, the X-ray photoelectron spectroscopy (XPS) survey spectrum is presented in Figure 2e, providing additional information on the elemental composition of the NPs.

**Figure 3:**
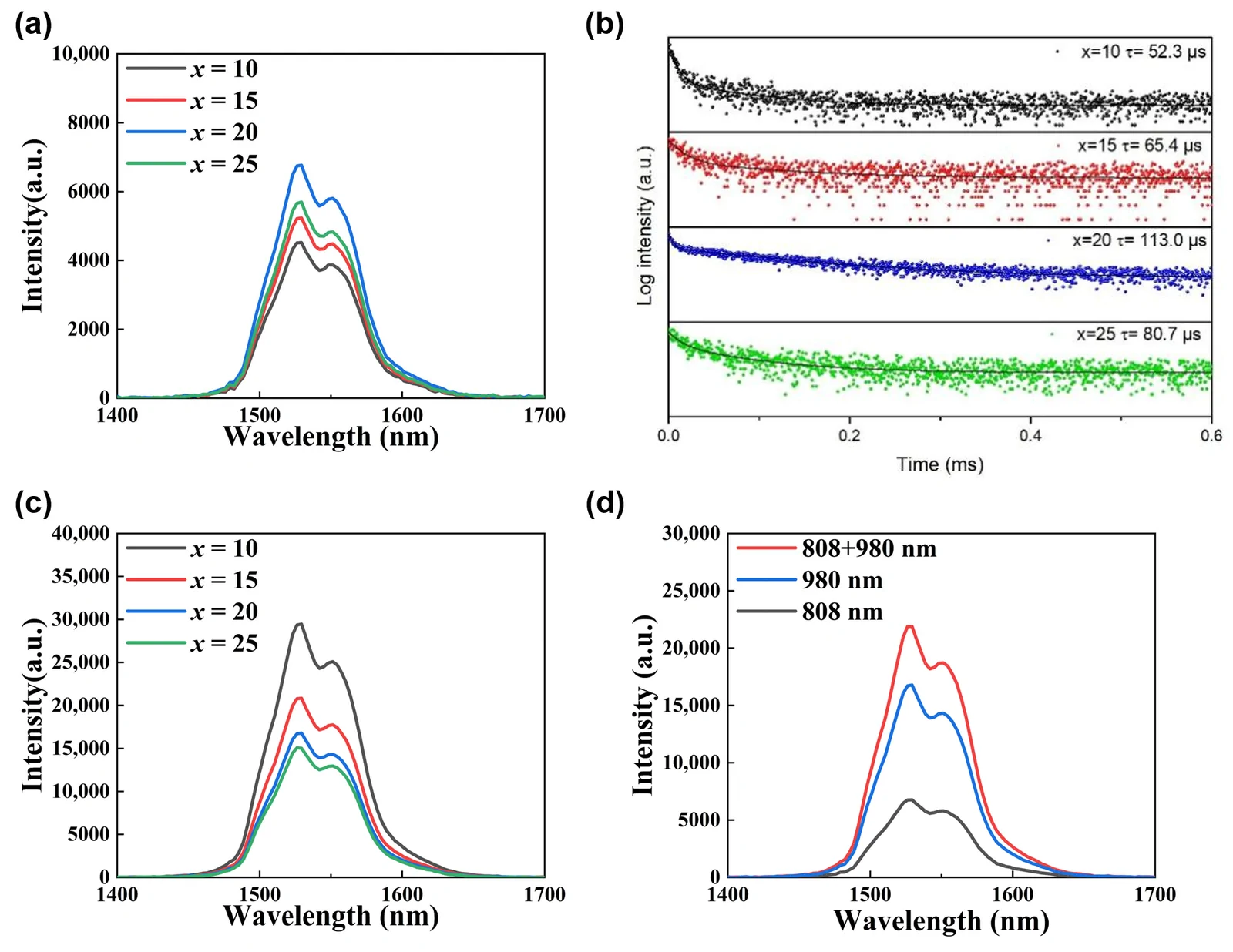
(**a**) Downconversion (DC) emission spectra of NaYbF_4_:2%Er^3+^, 2%Ce^3+^@NaYF_4_:20%Yb^3+^, *x*%Nd^3+^ (*x* = 10, 15, 20, and 25) core-shell NPs under 808 nm laser excitation. (**b**) Luminescence decay profiles of these NPs under 808 nm excitation, monitored at 880 nm. (**c**) DC emission spectra of these NPs under 980 nm laser excitation. (**d**) DC emission spectra of NaYbF_4_:2%Er^3+^, 2%Ce^3+^@NaYF_4_:20%Yb^3+^, 20%Nd^3+^ core-shell NPs under 808 nm, 980 nm, and simultaneous dual-wavelength (808 and 980 nm) excitation.

All NPs used for the DC measurements in Figure 3a,c,d were dispersed in cyclohexane at an NP concentration of 40 mg mL^−1^. Single-wavelength DC measurements were performed under either 808 nm or 980 nm excitation at a laser power of 75 mW, with a laser spot area of approximately 1 mm^2^, corresponding to a power density of 7.5 W cm^−2^. The DC emission spectra of the NPs under 808 nm excitation are presented in Figure 3a. The DC emission intensity initially increases with Nd^3+^ concentration, reaches its highest value at 20% Nd^3+^, and then decreases at 25%. The initial enhancement is consistent with increased Nd^3+^-mediated excitation through energy transfer from Nd^3+^ to Yb^3+^. At higher doping levels, cross-relaxation among neighboring Nd^3+^ ions may become more pronounced, contributing to concentration quenching and reduced emission intensity. To further examine the effect of Nd^3+^ concentration on sensitizer dynamics, time-resolved photoluminescence measurements of the Nd^3+ 4^F_3/2_ → ^4^I_9/2_ emission were performed under 808 nm excitation, with detection at 880 nm, as shown in Figure 3b. The measured lifetimes were 52.3, 65.4, 113.0, and 80.7 μs for the NPs containing 10%, 15%, 20%, and 25% Nd^3+^, respectively. Under 980 nm excitation, the DC emission intensity decreases monotonically with increasing Nd^3+^ concentration, as shown in Figure 3c. Because 980 nm light primarily excites Yb^3+^, this decrease may be associated with competing energy-transfer pathways, including Yb^3+^-to-Nd^3+^ back transfer, that reduce the excitation reaching Er^3+^. Considering the results of steady-state and time-resolved photoluminescence measurements, the 20% Nd^3+^ NPs was selected for further investigation. Among the investigated samples, these NPs exhibited the strongest Er^3+^ DC emission under 808 nm excitation and the longest Nd^3+^ luminescence lifetime, while retaining Er^3+^ DC emission under 980 nm excitation. Using the selected 20% Nd^3+^ NPs, we then compared the Er^3+^ DC luminescence under 808 nm, 980 nm, and dual-wavelength excitation. Under dual-wavelength excitation, the 808 nm and 980 nm lasers each provided 75 mW, giving a total excitation power of 150 mW. As shown in Figure 3d, dual-wavelength excitation produced stronger DC emission than either single-wavelength excitation under the measurement conditions. Under 808 nm excitation, Nd^3+^ absorbs the pump light through the ^4^I_9/2_ → ^4^F_5/2_ transition and then relaxes nonradiatively to the ^4^F_3/2_ level. Energy transfer from Nd^3+^ populates the Yb^3+ 2^F_5/2_ level, followed by energy transfer from Yb^3+^ to Er^3+^. Under 980 nm excitation, Yb^3+^ is directly excited through the ^2^F_7/2_ → ^2^F_5/2_ transition and subsequently transfers energy to Er^3+^. Dual-wavelength excitation accesses both the Nd^3+^ → Yb^3+^ → Er^3+^ and Yb^3+^ → Er^3+^ pathways, consistent with the observed increase in emission around 1.5 μm.

**Figure 4:**
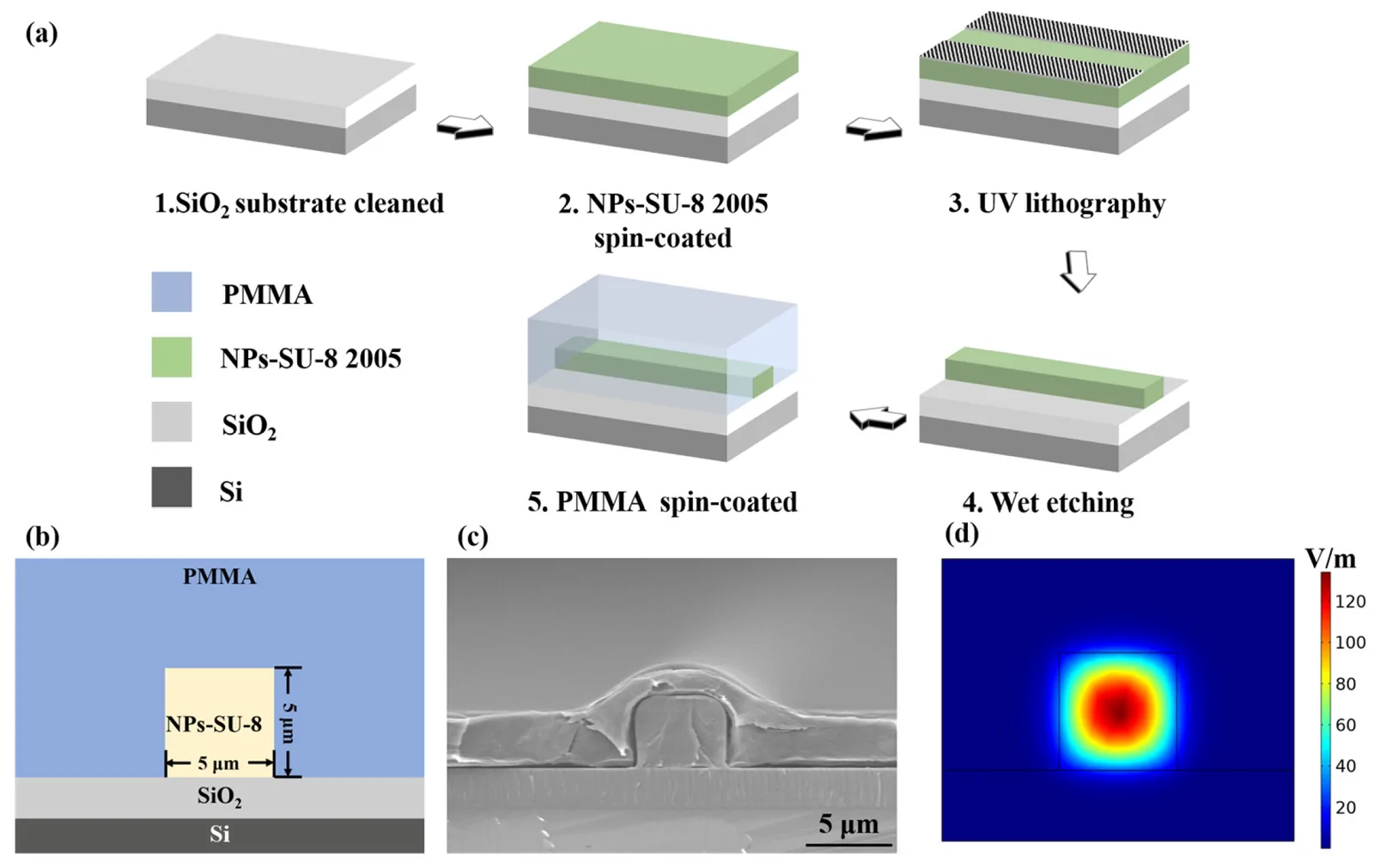
(**a**) Fabrication process of the polymer waveguide amplifier. (**b**) Schematic cross-sectional structure of the waveguide amplifier. (**c**) SEM image of the waveguide cross section, showing the core and cladding layers. (**d**) Simulated optical field distribution at 1535 nm, illustrating mode confinement within the waveguide core.

We further incorporated the as-prepared NaYbF_4_:2%Er^3+^, 2%Ce^3+^@NaYF_4_:20%Yb^3+^, 20%Nd^3+^ NPs into SU-8 2005 photoresist at a concentration of 2.46 wt%. The NPs were uniformly dispersed in the photoresist, forming an NPs-SU-8 2005 nanocomposite that served as the active core material of the waveguide amplifier. Here, SU-8 2005 is a commercial high-contrast, epoxy-based negative photoresist, with “2005” denoting a specific formulation within the SU-8 2000 series. It serves as the polymer matrix, while the dispersed rare-earth-doped NPs provide the active gain component. For optical confinement, a 2-μm-thick polymeric methyl methacrylate (PMMA) layer was used as the upper cladding. The SiO_2_ layer on the Si substrate served as the lower cladding and had a thickness of 3 μm with a thickness tolerance of ±5%. The underlying Si substrate had a thickness of 525 ± 10 μm. The measured refractive indices at 1535 nm were 1.481 for PMMA, 1.562 for the NPs-SU-8 2005, and 1.445 for SiO_2_, providing sufficient refractive index contrast for effective light guidance. The fabrication sequence of the rectangular waveguide amplifier is illustrated in Figure 4a. The NPs-SU-8 2005 nanocomposite was spin-coated onto a cleaned silica-on-silicon substrate using a two-stage process. Specifically, the mixture was first spin-coated at 500 rpm for 20 s to promote uniform spreading, followed by 3000 rpm for 20 s. This procedure produced a 5-μm-thick active core layer. After alignment of the chromium photomask, the core layer was exposed using the ABM/6/350 photolithography system under 365 nm i-line UV illumination. The exposure irradiance was 15 mW/cm^2^, and the exposure time was 7 s, corresponding to a dose of 105 mJ/cm^2^ selected with reference to the SU-8 processing guidelines. Exposure dose influences subsequent resist cross-linking and therefore affects pattern retention and linewidth after development. The chromium photomask provided UV contrast for selective exposure, and its straight stripe pattern was designed to form a 5-μm-wide waveguide. The designed core cross-section of 5 μm × 5 μm was selected with optical mode confinement and fiber coupling as design considerations. After exposure and post-exposure baking, the sample was developed in propylene glycol monomethyl ether acetate (PGMEA). The developer selectively dissolved and removed the unexposed SU-8, leaving the cross-linked exposed regions as the patterned waveguide core. The developed structure was then rinsed with isopropyl alcohol (IPA) and dried, followed by spin-coating a PMMA upper cladding layer to complete the waveguide amplifier structure. The cross-sectional schematic of the fabricated waveguide amplifier and the corresponding scanning electron microscopy (SEM) image are shown in Figure 4b,c, respectively. The optical field distribution at 1535 nm was simulated using COMSOL Multiphysics 6.0 based on the finite element method (FEM). A two-dimensional model of the waveguide cross-section was constructed using the refractive indices reported above. The guided modes were calculated using the Mode Analysis study in the Electromagnetic Waves, Frequency Domain (ewfd) physics interface. The fundamental guided mode was selected to obtain the optical field distribution shown in Figure 4d. The simulation confirms strong confinement of the 1535 nm signal light within the core region, which is essential for achieving high amplifier gain.

**Figure 5:**
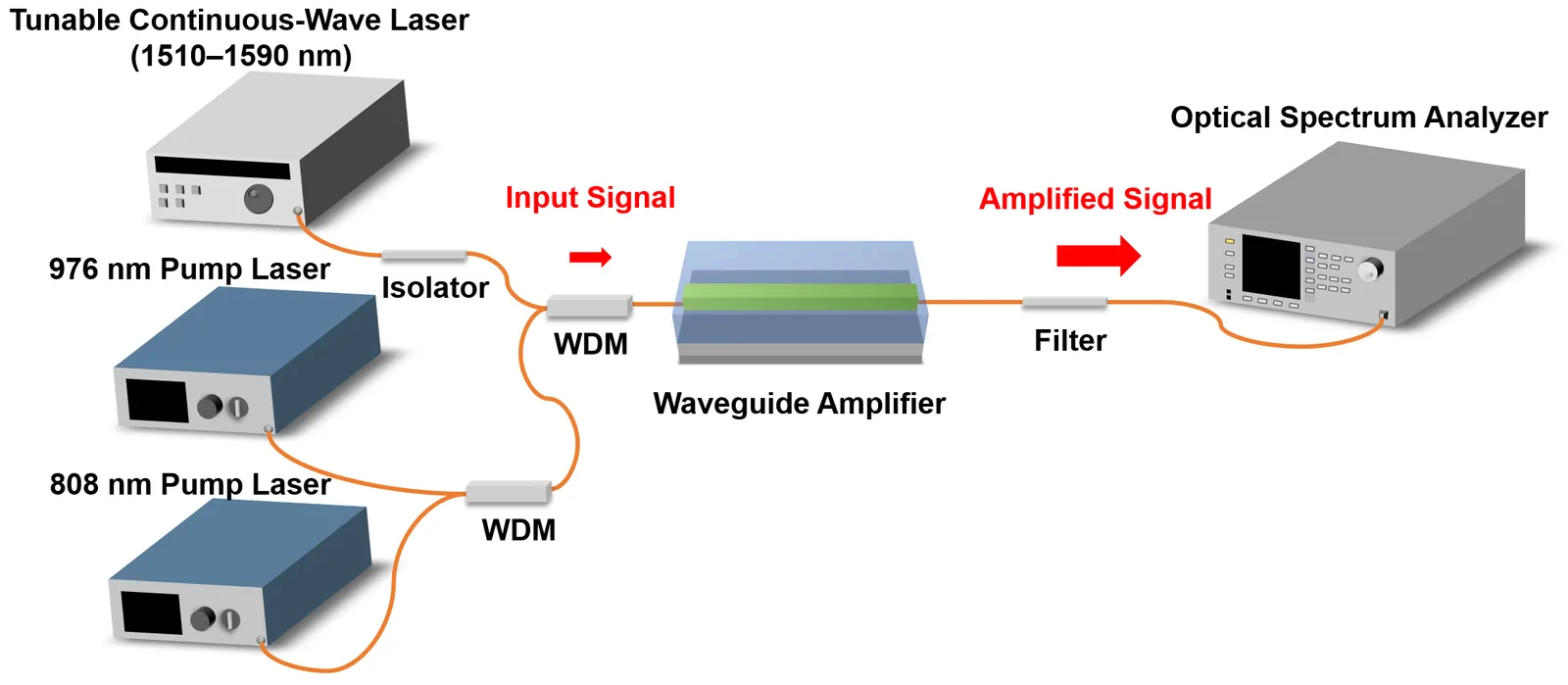
Experimental setup for measuring the gain performance of a C-band polymer optical waveguide amplifier, illustrating the 808 and 976 nm pump lasers, C-band signal laser, and optical spectrum acquisition system.

The optical amplification performance of the waveguide amplifier was evaluated using the experimental setup illustrated in Figure 5. The measurement system employed two wavelength-division multiplexers (WDMs) to integrate multiple optical paths. A tunable semiconductor laser (Santec TSL-210, Komaki, Japan) operating in the 1510–1590 nm range served as the signal source, while continuous-wave laser diodes at 808 nm (AS-SPL-808-250-FA-B, Shanghai Aoshow Information, Shanghai, China) and 976 nm (AS-SPL-976-1000-FA-B, Shanghai Aoshow Information, Shanghai, China) were used as pump sources. An optical isolator was inserted between the signal source and the waveguide input to suppress self-excited oscillations and minimize feedback, thereby enhancing system stability. The amplified output signal was collected from the waveguide facet via a single-mode fiber and analyzed using an optical spectrum analyzer (MS9740, Anritsu, Atsugi, Japan).

**Figure 6:**
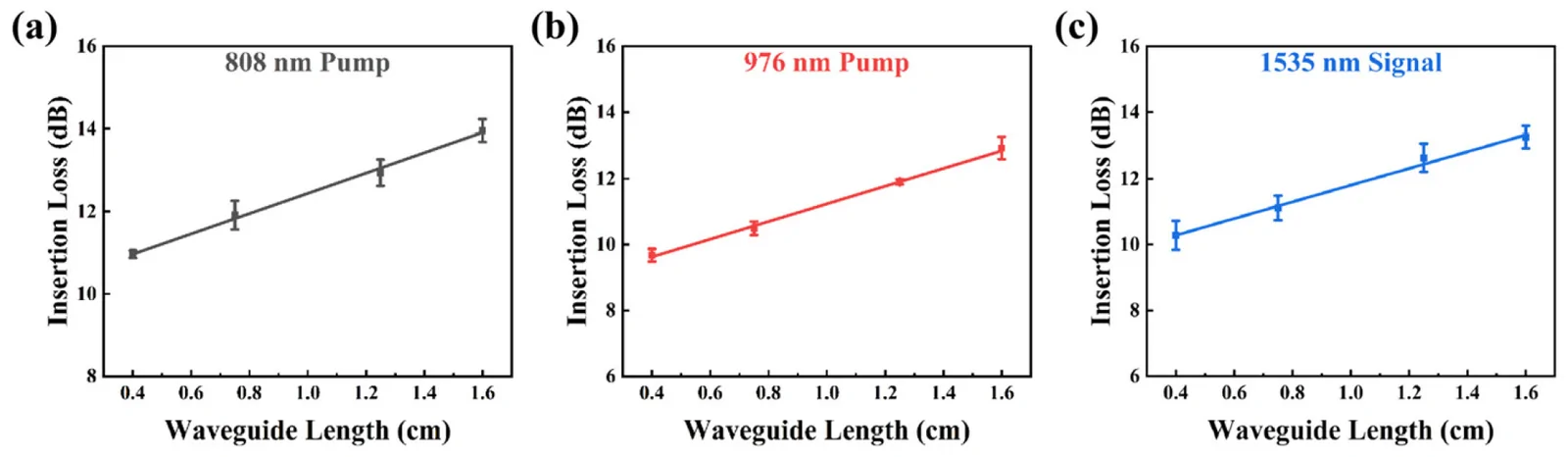
Length-dependent insertion loss of the waveguide amplifier and corresponding fitted curves at (**a**) 808 nm, (**b**) 976 nm, and (**c**) 1535 nm.

Prior to the gain measurements, the insertion losses (ILs) at the pump and signal wavelengths were measured to determine the propagation and coupling losses and to calibrate the corresponding on-chip powers. The IL at each wavelength is defined as:

(1) $$\mathrm{Insertion Loss}=10\log_{10}\left(\frac{P_{\mathrm{in}}}{P_{\mathrm{out}}}\right)$$

where *P_in_* and *P_out_* are the optical powers measured before input coupling and after output coupling, respectively. The same definition was applied independently at the pump wavelengths of 808 and 976 nm and the signal wavelength of 1535 nm. The measured IL comprises the fiber-to-waveguide coupling losses at the input and output facets and the propagation loss within the waveguide. The cut-back method [18,29] was implemented by measuring the ILs of waveguides with different lengths. The measured IL was linearly fitted according to:

(2) Insertion Loss = *α_p_L* + 2*α_c_*

where *L* is the waveguide length in cm, *α_p_* is the propagation loss in dB/cm, and *α_c_* is the coupling loss per facet in dB. Thus, the slope of the fitted line gives the propagation-loss, while the intercept, 2*α_c_*, represents the total coupling loss of the two facets. As shown in Figure 6a–c, the propagation losses were determined to be 2.46 ± 0.07 dB/cm at 808 nm, 2.68 ± 0.07 dB/cm at 976 nm, and 2.54 ± 0.16 dB/cm at 1535 nm. The corresponding coupling losses were 4.99 dB/facet, 4.28 dB/facet, and 4.63 dB/facet at 808 nm, 976 nm, and 1535 nm, respectively. These coupling losses are primarily attributed to imperfections in the waveguide end facets and mode mismatch between the optical fiber and the waveguide. Based on these loss measurements, both the pump and signal powers were calibrated to the on-chip power levels.

**Figure 7:**
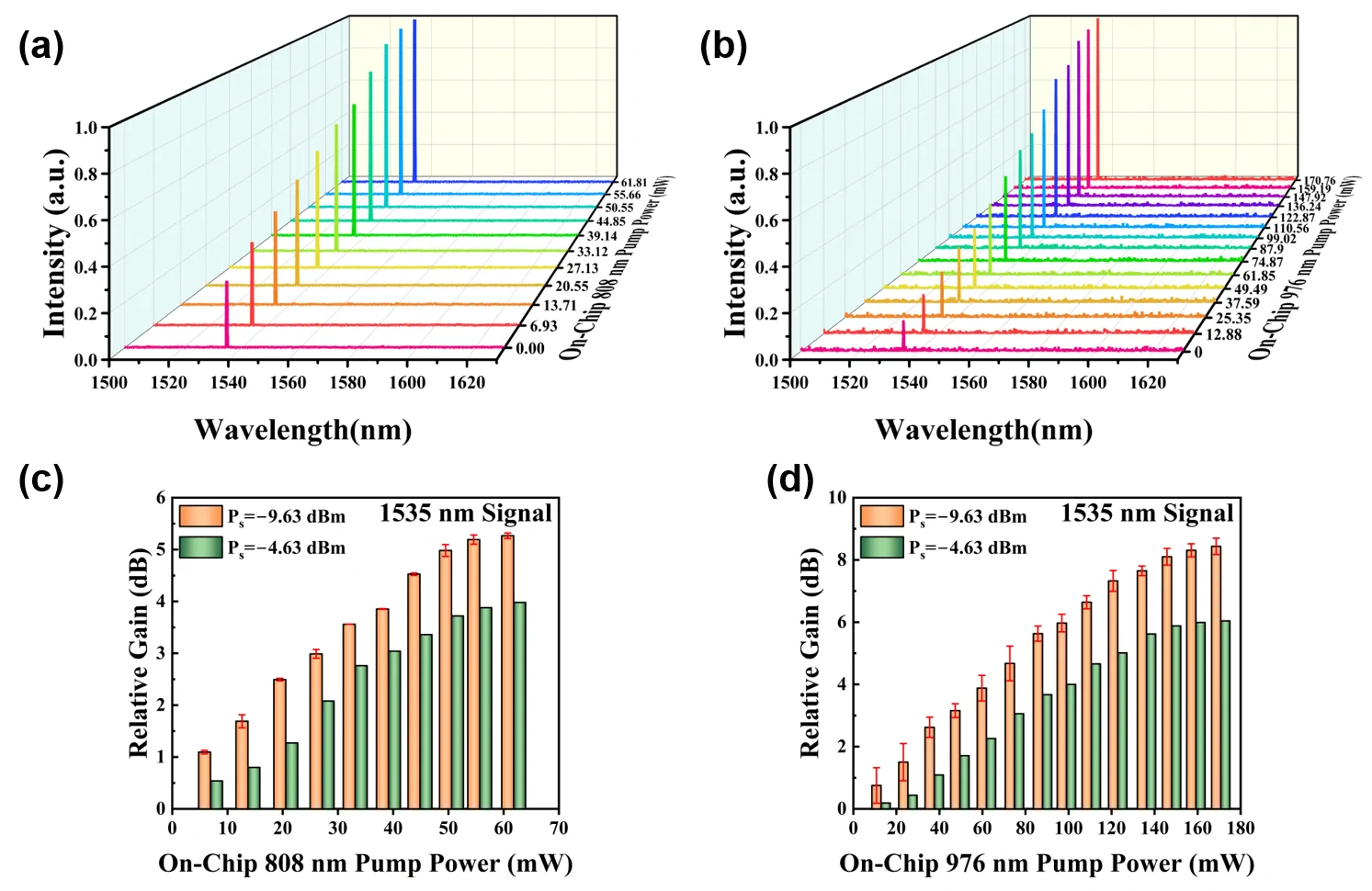
Output signal spectra at 1535 nm as a function of (**a**) on-chip single 808 nm pump power and (**b**) on-chip single 976 nm pump power at a signal power of −9.63 dBm. Relative gain as a function of pump power for different signal powers with a signal wavelength of 1535 nm under single-laser excitation, (**c**) 808 nm pump and (**d**) 976 nm pump.

The relative gain of the waveguide amplifier is defined as:

(3) $$\mathrm{Relative Gain}=10\log_{10}\left(\frac{P_{s}^{\mathrm{on}}}{P_{s}^{\mathrm{off}}}\right)$$

where $P_{s}^{\mathrm{on}}$ and $P_{s}^{\mathrm{off}}$ represent the output signal powers measured with and without the pump laser excitation, respectively. Figure 7a,b display the output signal spectra under 808 nm and 976 nm excitation, respectively, with the signal power at −9.63 dBm. As the pump power increases, the output signal intensity rises correspondingly, indicating effective optical amplification. Figure 7c,d illustrate the relationship between the relative gain and varying signal power at a signal wavelength of 1535 nm. Under 808 nm (61.81 mW) and 976 nm (170.76 mW) pump laser excitation, the relative gain reached 5.27 ± 0.05 dB and 8.44 ± 0.26 dB, respectively, in the 0.8 cm long rectangular waveguide. For a waveguide core cross-sectional area of 5 μm × 5 μm, these on-chip pump powers correspond to input pump power densities of approximately 2.47 and 6.83 mW/μm^2^, respectively. For a fixed pump power, the relative gain achieved with 976 nm pumping is higher than that obtained with 808 nm pumping, which aligns with the trend observed in the DC luminescence of as-prepared NaYbF_4_:2%Er^3+^, 2%Ce^3+^@NaYF_4_:20%Yb^3+^, 20%Nd^3+^ NPs. At on-chip signal powers of −9.63 dBm and −4.63 dBm, the corresponding relative gains were measured to be 8.44 ± 0.26 dB and 6.04 dB, respectively. This observed decrease in gain with increasing input signal power is attributed to a reduction in population inversion between the ^4^I_13/2_ and ^4^I_15/2_ levels of Er^3+^ at higher signal intensities, which in turn limits the maximum achievable gain.

**Figure 8:**
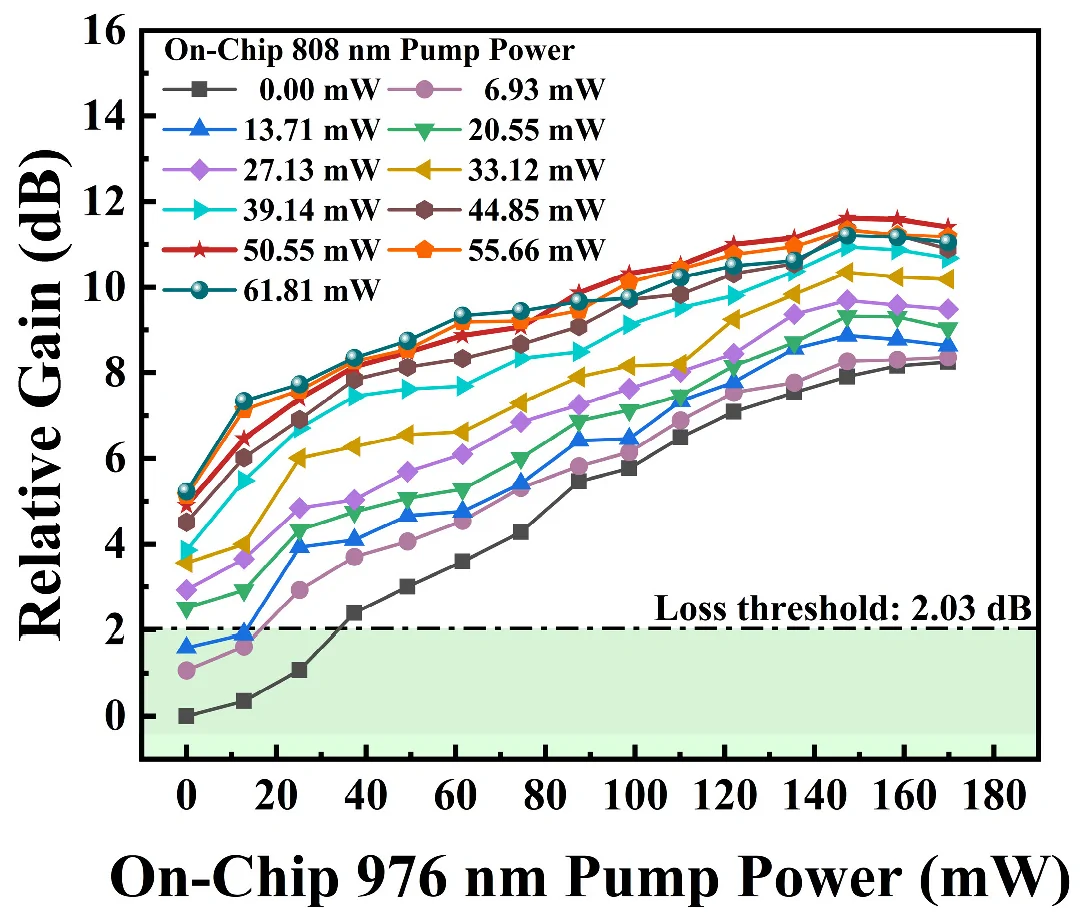
Relative gains of the fabricated waveguide amplifier as a function of the on-chip 976 nm pump power at different fixed on-chip 808 nm pump powers.

To explore the effect of dual wavelength pumping, we evaluated the gain performance of the fabricated waveguide amplifier under 808 nm and 976 nm excitation at a signal wavelength of 1535 nm and an on-chip signal power of −9.63 dBm. Figure 8 shows the relative gain as a function of the on-chip 976 nm pump power, with each curve corresponding to a fixed on-chip 808 nm pump power. As the 976 nm pump power increases, the relative gain gradually increases and approaches saturation. Among the pump combinations tested, on-chip powers of 50.55 mW at 808 nm and 147.33 mW at 976 nm yielded the highest measured relative gain of 11.62 dB. These on-chip pump powers correspond to input pump power densities of approximately 2.02 and 5.89 mW/μm^2^, respectively. For comparison, the highest relative gains under 808 nm-only and 976 nm-only pumping were 5.27 ± 0.05 dB at 61.81 mW and 8.44 ± 0.26 dB at 170.76 mW, respectively. This gain was 6.35 dB higher than the highest gain under 808 nm-only pumping and 3.18 dB higher than that under 976 nm-only pumping. The additional 808 nm excitation activates the Nd^3+^ → Yb^3+^ → Er^3+^ energy-transfer pathway alongside the Yb^3+^ → Er^3+^ pathway excited by the 976 nm pump, contributing to the observed gain enhancement. To further evaluate the amplification capability of the waveguide amplifier, the internal net gain was calculated by subtracting the propagation loss of the waveguide from the measured relative gain [30], according to:

(4) $$\mathrm{Internal Net Gain}=10\log_{10}\left(\frac{P_{s}^{\mathrm{on}}}{P_{s}^{\mathrm{off}}}\right)-\alpha_{p}L$$

where the first term is the measured relative gain, *α_p_* is the propagation loss at 1535 nm obtained from Figure 6c, and *L* is the waveguide length. This parameter represents the signal power enhancement through the waveguide after accounting for propagation loss, excluding input and output coupling losses. For the 0.8 cm waveguide, the measured propagation loss of 2.54 dB/cm corresponds to a propagation loss of approximately 2.03 dB, indicated by the dashed line in Figure 8. Based on the measured propagation loss and waveguide length, the internal net gain under dual-wavelength pumping was calculated to be 9.59 dB.

To further evaluate the performance of the fabricated polymer waveguide amplifier and highlight the proposed gain-enhancement strategy, a comparison with previously reported polymer waveguide amplifiers based on rare-earth-doped NPs is summarized in Table 1. The table summarizes the key performance parameters and experimental conditions. Our work introduces Nd^3+^-sensitized core-shell NPs as the gain medium, providing an additional 808 nm excitation pathway for dual-wavelength pumping. Under the tested conditions, the highest measured relative gain at 1535 nm under combined 808 and 976 nm pumping exceeded that obtained under 976 nm-only pumping.

## 3. Conclusions

In conclusion, we have introduced a gain-enhancement strategy for C-band waveguide amplifiers by using core-shell NPs co-doped with multiple rare-earth ions (NaYbF_4_:Er^3+^, Ce^3+^@NaYF_4_:Yb^3+^, Nd^3+^). Under dual-wavelength (808 and 976 nm) pumping, the 0.8-cm-long polymer waveguide amplifier achieved a maximum measured relative gain of 11.62 dB at 1535 nm, which is 3.18 dB higher than the highest mean relative gain obtained under 976 nm single-wavelength pumping. Introducing Nd^3+^ sensitization provides an additional 808 nm excitation pathway that can be combined with Yb^3+^-mediated excitation under 976 nm pumping for C-band amplification. This dual-wavelength pumping strategy offers an approach to designing compact photonic devices with potential applications in PICs used in telecommunications. With further optimization nanocomposite and waveguide design, this approach may improve amplification performance and facilitate integration into photonic circuits.

## Figures and Tables

**Table 1:** Comparison of polymer waveguide amplifiers based on rare-earth-doped nanoparticles.

Gain Medium	Matrix	Waveguide Type	λ_s_ (nm)	λ_P_ (nm)	Signal Power (dBm)	Pump Power (mW)	Length (cm)	Relative Gain (dB)	Reference
NaYF_4_:Er^3+^, Yb^3+^, Ce^3+^	SU-8	Rectangular	1535	976	−10	210	1.3	4.0	[26]
NaYF_4_:Er^3+^, Yb^3+^	PMMA	Groove	1540	976	−8.54	400	1.5	7.6	[27]
NaYF_4_:Er^3+^, Yb^3+^	SU-8	Rectangular	1530	980	−10	270	0.9	5.6	[20]
NaYbF_4_:Er^3+^, Ce^3+^@NaYF_4_:Yb^3+^, Nd^3+^	SU-8	Rectangular	1535	808	−9.63	61.81	0.8	5.27	This work
				976		170.76		8.44	
				808 + 976		50.55 + 147.33		11.62	

## Data Availability

The data that support the findings of this study are available upon request from the authors.
